# KTt-45, a T-type calcium channel blocker, acts as an anticancer agent by inducing apoptosis on HeLa cervical cancer cell line

**DOI:** 10.1038/s41598-023-47199-1

**Published:** 2023-12-13

**Authors:** Nguyen Huy Du, Truong Thi Bich Ngoc, Huynh Qui Cang, Nguyen Thi Thuy Luyen, Tran Linh Thuoc, Tran Le Quan, Dang Thi Phuong Thao

**Affiliations:** 1 Department of Molecular and Environmental Biotechnology, Faculty of Biology and Biotechnology, VNU-HCM, University of Science, 227 Nguyen Van Cu, Ho Chi Minh City, 700000 Vietnam; 2 Laboratory of Cancer Research, VNU-HCM, University of Science, Duong so 4, Linh Trung, Thu Duc, Ho Chi Minh City, 700000 Vietnam; 3 Vietnam National University, Ho Chi Minh City, Vo Truong Toan, Linh Trung, Thu Duc, Ho Chi Minh City, 700000 Vietnam; 4 Central Laboratory of Analysis, VNU-HCM, University of Science, 227 Nguyen Van Cu, Ho Chi Minh City, 700000 Vietnam; 5 Department of Hydro-Geology-Engineering Geology and Environmental Geology, Faculty of Geology, VNU-HCM, University of Science, 227 Nguyen Van Cu, Ho Chi Minh City, 700000 Vietnam; 6 Department of Medicinal Chemistry, Faculty of Chemistry, VNU-HCM, University of Science, 227 Nguyen Van Cu, Ho Chi Minh City, 700000 Vietnam

**Keywords:** Drug discovery, Cancer, Cancer therapy, Drug development

## Abstract

The abnormal expression in the T-type calcium channels is involved in various cancer types, thus inhibiting T-type calcium channels is one of approaches in cancer treatment. The fact that KTt-45 acted as a T-type calcium channel inhibitor as well as a pain-relief agent prompts us to address if KTt-45 plays any role against cancer cells. The results showed that KTt-45 caused cytotoxic effects towards HeLa cervical, Raji lymphoma, MCF-7 breast cancer, and A549 lung cancer cell lines with IC_50_ values less than 100 μM, in which highly selective toxicity was against HeLa cells (IC_50_ = 37.4 μM, SI > 3.2). Strikingly, the KTt-45 induced an accumulation of cytoplasmic vacuoles after 48 h treatment and mitochondrial-dependent apoptosis activation as evidenced by morphological features, chromatin condensation, nuclear fragmentation, and significant activation of caspase-9 as well as caspase-3. In conclusion, KTt-45 could inhibit cell growth and trigger mitochondrial-dependent apoptosis in HeLa cervical cancer cells. The results, taken together, strongly demonstrated that KTt-45 is a potential agent for further study on anticancer drug development which not only targets cancer cells but also helps to relieve neuropathic pain in cancer patients.

## Introduction

Cancer is one of the most leading causes of death worldwide. In the year 2020, 19.3 million newly diagnosed cases and nearly 10 million death cases were recorded around the world^1^, which is not only a burden on society but also a challenge that modern medicine needs to solve. In the present, the most common cancers were recorded namely breast (11.7%), lung (11.4%), colorectal (10.0%), prostate (7.3%), stomach (5.6%), liver (4.7%), and cervix uteri (3.1%)^1^. Based on GLOBOCAN’s prediction, the number of cancer cases will increase to 28.4 million in 2040^1^. In terms of cancer treatment, surgery, radiotherapy, and chemotherapy are usually applied depending on cancer types and disease progress. Additionally, recently, various therapies including immunotherapy, and targeted biological therapy are also utilized in combination to enhance treatment efficacy. However, the cancer death rate still remains high due to late diagnosis, drug resistance, tumor relapse, and metastasis^2^. Therefore, relentless attempt in current research aims to discover novel anticancer agents in order to overcome these challenges and simultaneously improve the life expectancy of patients.

6-prenylnaringenin (6-PNG) compound has been reported on anticancer activity in previous studies. Particularly, 6-PNG exhibited cytotoxicity towards colorectal, ovarian, breast, prostate adenocarcinoma, and melanoma cancer cell lines^3–6^. Regarding the mechanism of 6-PNG, non-apoptotic cell deaths were induced in prostate cancer and melanoma cell lines^6,7^, and the inhibition of histone deacetylase caused by 6-PNG also contributed to the anticancer ability of this compound^6^. In our work, 6-(3-ethylpent-2-enyl)-5,7-dihydroxy-2-(2-hydroxyphenyl)chroman-4-one (KTt-45) was synthesized based on the structure of 6-prenylnaringenin (6-PNG) with some modifications^8^, could be a promising candidate for anticancer investigation.

Notably, KTt-45 has been identified as a novel blocker of T-type calcium channels in a recent report^8^. Calcium signaling plays an important role in the regulation of several cellular processes including cell cycle^9^, proliferation^10^, survival^11^, apoptosis^11^, motility^12^, exocytosis^13^, endocytosis^13^, and also contributes to tumor growth^14^. To be more specific, intracellular Ca^2+^ could activate calmodulin, thereby controlling the expression of different cyclin-dependent kinases which regulate cell cycle and proliferation. The entry of extracellular calcium is controlled by calcium channels, in which voltage-activated Ca^2+^ channel (VA) is one of calcium channels allowing calcium entry dependent on membrane potential in response to the depletion of intracellular calcium store. VA is divided into high-voltage activated Ca^2+^ channel (HVA) (L, P/Q, N, R-type channels) and low-voltage activated Ca^2+^ channel (LVA) (T-type channel)^15^. The distinct properties of T-type Ca^2+^ channels including activation at low-voltage and transient kinetics of inactivation produce oscillatory Ca^2+^ waves which are important for cell cycle progression^16^. Especially, T-type channels have been considered as potential targets in cancer treatment due to their overexpression in diverse cancer types when compared with normal tissues including breast, colorectal, prostate, leukemia, ovary, melanoma, and liver cancer^9,17^. Some T-type Ca^2+^ channel blockers including mibefradil, NNC-55-0396, KYS05090 and its derivatives exhibited anticancer property due to inducing the ROS accumulation^18^, ER stress^19^, cell cycle arrest^20^, apoptotic activation^18,20,21^, and autophagy inhibition^18,19^. Strikingly, mibefradil was applied in a Phase 1b clinical trial (ClinicalTrials.gov identifier: NCT01480050), in which mibefradil would be administrated for glioma patients prior to treating with chemotherapeutic agent temolozomide in order to enhance the efficacy. In addition, a T-type calcium channel inhibitor TTA-A2 when used as a single agent or in a combination with paclitaxel could inhibit the growth, viability, and metastasis of A549 lung adenocarcinoma cells and 3D spheroids of this cell line^22^. Therefore, the anticancer capacity of KTt-45 compound, a novel T-channel blocker, also needs to be elucidated.

Although KTt-45 compound exhibited as a potent pain-reliever for neuropathic pain induced by chemotherapeutic drug bortezomib in a previous report^8,23^, the anticancer activity of this compound has not been studied yet. If utilized, this compound could be used in combination with clinical drugs to not only target cancer cells but also reduce symptoms of neuropathic pain in cancer patients. For the above reason, in this study, the anticancer activity of KTt-45 was explored by investigating cytotoxic effect on four human cancer cell lines, and the molecular mechanism of KTt-45’s impacts was also initially elucidated.

## Materials and methods

### Cell lines and reagents

In this study, five human cell lines were utilized including A549 (lung carcinoma, ATCC CCL-185), HeLa (cervical carcinoma, ATCC CCL-2), MCF-7 (breast adenocarcinoma, ATCC HTB-22), Raji (Burkitt’s lymphoma, ATCC CCL-86), and BJ-5ta (human normal fibroblasts, ATCC CRL-4001). A549 cells were cultured using DMEM-F12 medium (Himedia, Indian). HeLa cell line was cultured in EMEM medium (Himedia, India), while MCF-7 and BJ-5ta cells were cultured in high glucose DMEM medium (Sigma-aldrich, Germany). Raji cells were cultured using RPMI medium (Himedia, India). The culture mediums were supplemented with 10% fetal bovine serum (FBS) (Sigma-aldrich, Germany). The cell lines were cultured in 37 °C incubator with humidified 5% CO_2_ atmosphere, and the medium was renewed every 2–3 days.

Dimethyl sulfoxide (DMSO) was obtained from Sigma-alrich (Germany), stored at room temperature. Trypsin, MTT (3-(4,5-Dimethylthiazol-2-yl)-2,5-diphenyltetrazolium bromide) were supplied by Sigma-aldrich (Germany), stored at 4 °C temperature. Doxorubicin was supplied by Ebewe Pharma (Austria), preserved at 4 °C temperature. Paraformaldehyde was obtained from Scharlau (Spain), and Triton-X100 was purchased from Pancreac AppliChem (Spain-Germany-Italia), kept at room temperature. Monodansylcadaverine (MDC) was purchased from Santa Cruz Biotechnology (USA), maintained at − 30 °C temperature. Anti cleaved-caspase-3 antibody (#9661), anti cleaved-caspase-9 antibody (#20750), anti LC3A/B antibody (#4108S) were purchased from Cell Signaling Technology (USA), stored at − 30 °C temperature. Anti-rabbit secondary antibody (#A-11008) was purchased from Invitrogen (USA), stored at − 30 °C temperature. Normal goat serum (NGS) was purchased from Vector Laboratories (Japan), preserved at − 30 °C temperature. FITC Annexin-V Apoptosis Detection Kit I (#556547) was obtained from BD Biosciences (USA). TriSure, Tetro cDNA Synthesis Kit, and SensiFAST™ HRM Realtime PCR Kit were supplied by Bioline (UK). qPCR primers were purchased from PhuSa (Vietnam). The qRT-PCR reagents were stored at − 30 °C temperature, while TriSure kit was kept at 4 °C temperature. KTt-45 (6-(3-ethylpent-2-enyl)-5,7-dihydroxy-2-(2-hydroxyphenyl)chroman-4-one) was synthesized in-house as reported previously by Nguyen et al.^8^, then stored at − 30 °C.

### Preparation of KTt-45 compound

The KTt-45 compound was dissolved in 99.9% (v/v) DMSO to gain stock solution at concentration of 120 mM, which was preserved at − 30 °C until experiments. The experimental design was depicted in Supplementary Fig. S1. For cytotoxicity assay, the stock solution was diluted in culture medium to reach experimental concentrations ranging from 0.5 to 120 μM immediately before each experiment.

### Cytotoxicity assay

The adherent cells were detached from culture dishes by 0.25% trypsin-0.53 mM EDTA, centrifuged to collect cell pellet, then cell density was identified by Neubauer chamber (Hirschmann, Germany). After that, 2 × 10^4^ cells/100 μl were transferred into each well of 96-well plate, incubated overnight for the adhesion. The day after, the culture medium was discarded, replaced by 100 μl of medium containing the compound at experimental concentrations. Meanwhile, for Raji suspension cells, 2 × 10^4^ cells/50 μl were added into wells, incubated within 1 h for the reconstitution, then 50 μl of medium including the compound was supplemented. To investigate the cytotoxicity of KTt-45, the concentrations ranging from 0.5 to 120 μM were utilized, and the final concentration of DMSO was kept less than 0.1% (v/v) in each well. Negative control sample was treated with 0.1% (v/v) DMSO. Blank wells contained medium with the compound or 0.1% (v/v) DMSO. Next, 96-well plates were incubated for 48 h before being subjected to MTT assay.

Regarding MTT assay, each of experimental wells was received 5 μl of 5 mg/ml MTT solution to gain the final MTT concentration of 0.25 mg/ml, incubated within 4 h. Subsequently, 60 μl of lysis buffer (30% (w/v) SDS, 0.03 N HCl) and 90 μl of DMSO (99.9% (v/v)) were added into wells to lyse cells and solubilize formazan crystals, then the plates were shaken at 750 rpm for 10 min. Finally, the absorbance was measured at wavelength of 550 nm by microplate reader (Thermofisher scientific, USA). The percentage growth inhibition at each concentration was calculated following the formula:

$$ {\text{I}}\% \, = \,\left( {{1}{-}\left( {{\text{OD}}_{{{\text{conc}}.}} {-}{\text{OD}}_{{{\text{blank}}}} } \right)/\left( {{\text{OD}}_{{{\text{ctrl}}}} {-}{\text{OD}}_{{{\text{blank}}}} } \right)} \right)\, \times \,{1}00\% . $$

The whole assay was performed at least in triplicate. The correlation between I% and compound concentrations was displayed by nonlinear sigmoidal curve established by Graphpad Prism software. Based on that, the IC_50_ value (the concentration causing 50% inhibition of cell growth) was interpolated. Besides, selectivity index (SI) of the compound towards a cancer cell line was also calculated by the ratio of IC_50_ on normal fibroblasts to IC_50_ on that cancer cell line.

### Assessment of KTt-45’s effect on cell growth during time

To further investigate the cytotoxicity of KTt-45, the effect on cell growth during time was assessed through OD_550nm_ values of MTT assay at different time points of 0 h, 24 h, 48 h, and 72 h. To be more specific, 2 × 10^4^ cells were seeded into each well and treated with KTt-45 compound at IC_50_ concentration. Negative controls and blanks were also established. The OD_550nm_ values were recorded at each time point. In addition, the changes in cell morphology were also captured using inverted microscope Nikon Eclipse TiU (Nikon, Japan) at the magnification of 400X.

### DAPI staining

The nuclear morphology of cells treated with KTt-45 was examined by DAPI staining. At first, 2 × 10^4^ cells were seeded into wells before being incubated with KTt-45 compound at IC_50_ concentration. Negative control sample was treated with 0.1% (v/v) DMSO. After the treatment, the cells were fixed by 10% (v/v) paraformaldehyde for 10 min, permeabilized by 0.1% (v/v) Triton-X100 for 5 min, and stained with 0.3 μM DAPI solution for 15 min. After being washed thrice with PBS solution, the cells were visualized under a fluorescence microscope (Nikon Eclipse TiU, Japan) at the magnification of 400X.

### Investigation of caspase 3/9 activation

The activation of caspase 3/9 inside KTt-45-treated cells was investigated by immunofluorescence staining. In the beginning, 2 × 10^4^ cells were transferred to each well and treated with KTt-45 compound at IC_50_ concentration. Then, similar to DAPI staining, cells were also fixed 10% paraformaldehyde for 10 min, permeabilized by 0.1% Triton-X100 for 5 min. Subsequently, cells were incubated with 10% (v/v) normal goat serum (NGS) for 30 min before an exposure to anti-cleaved caspase 3 (1:400 (v/v)) or anti-cleaved caspase 9 (1:800 (v/v)) antibody for 1 h. After being washed thrice with PBS solution, the co-incubation with secondary antibody (1:500 (v/v)) and 0.3 μM DAPI was conducted within the next hour. At last, after washing steps, the cells were observed under the fluorescence microscope.

### Annexin-V/PI staining

In this assay, 2 × 10^4^ cells were transferred to each well and treated with KTt-45 compound at IC_50_ concentration. After the treatment, cells were washed with cold PBS, incubating with annexin-V-FITC/PI binding buffer (1 μl annexin-V-FITC + 1 μl PI + 100 μl binding buffer 1X) for 15 min at room temperature in the dark, then being washed with PBS thrice before being visualized under fluorescence microscope.

### Monodansylcadaverine staining

Monodansylcadaverine (MDC) staining was used to detect autophagic vacuoles in cultured cells. In the same way, 2 × 10^4^ cells were transferred to each well and treated with KTt-45 compound at IC_50_ concentration. After the treatment, cells were incubated with 0.1 mM MDC at 37 °C for 15 min. Then, cells were washed thrice before being examined under the fluorescence microscope.

### Investigation of intracellular LC3

LC3 is considered as one of autophagic markers. The accumulation of LC3 inside KTt-45-treated cells was examined by immunofluorescence staining using anti-LC3 primary antibody (1:100 (v/v)). Similarly, 2 × 10^4^ cells were incubated with KTt-45 compound at IC_50_ concentration. The protocol was similar to aforementioned steps of caspase 3/9 investigation.

### Quantitative RT-PCR

After the treatment with KTt-45 compound at IC_50_ concentration, total RNA of samples were extracted by TriSure Kit following the manufacturer’s instructions. Next, 2 μg of total RNA was subjected to reverse transcription using Tetro cDNA synthesis kit and oligo-dT primers following the manufacturer’s protocol. After that, realtime PCR was performed with SensiFAST HRM Kit using 1 μl of cDNA template and final primers’ concentration of 500 nM. qPCR primer sequences were referred to previous documents^24–27^. *ATG5*-F: 5’-TGGATTTCGTTATATCCCCTTTAG-3’, *ATG5*-R: 5’-CCTAGTGTGTGCAACTGTCAA-3’, *ATG7*-F: 5’-TGAGTTGACCCAGAAGAAGCT-3’, *ATG7*-R: 5’-CCCAGCAGAGTCACCATTGT-3’, *LC3B*-F: 5’-AATCCCGGTGATAATAGAACGA-3’, *LC3B*-R: 5’-GGAGACGCTGACCATGCTGT-3’, *ACTB*-F: 5’-CATCGAGCACGGCATCGTCA-3’, *ACTB*-R: 5’-TAGCACAGCTGGATAGCACC-3’. The reference gene was *ACTB* (*β-actin*). Thermal cycling conditions were as follows: 1 cycle denaturation of 95 °C for 2 min, 45 cycles of 95 °C for 5 s, annealing temperature for 10 s, 72 °C for 10 s. The relative mRNA expression levels in treated samples were calculated by the formula 2^–ΔΔCt^.

### Statistical analysis

The experimental data were analyzed and graphed by Graphpad Prism 6 software. The results were expressed as mean ± SD. The mean differences between two groups were tested by unpaired Student’s *t* test (two-tailed), in which p-value ≤ 0.05 indicates a statistically significant difference. The non-linear regression curves displaying the correlation between I% values and logarithm of KTt-45 concentrations were created by Graphpad Prism, from which IC_50_ values were interpolated.

## Results

### Cytotoxicity of KTt-45 on cancer cell lines

**Figure 1:**
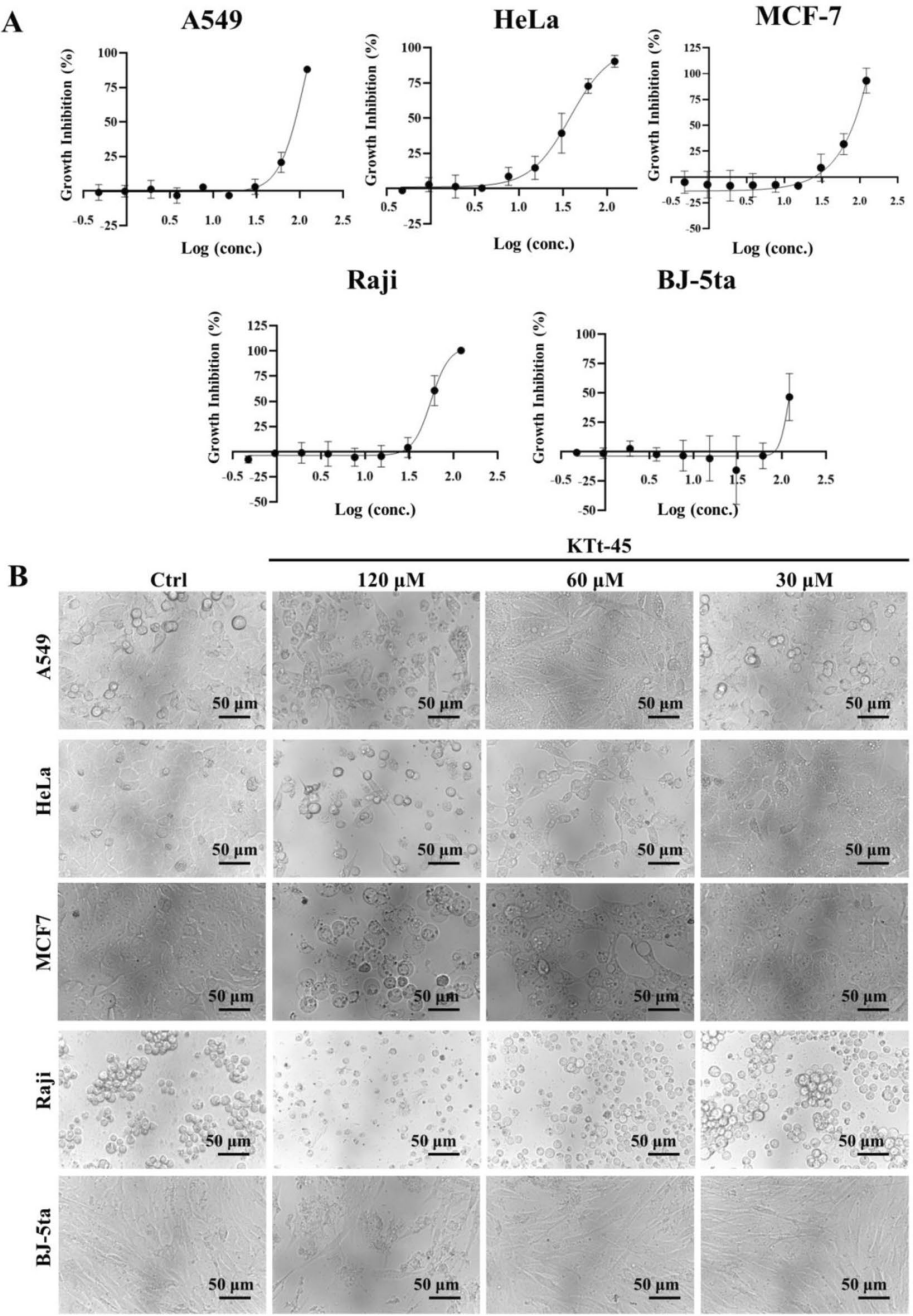
The cytotoxicity of KTt-45 on different cell lines. (**A**) The correlation between KTt-45 concentrations and percentage growth inhibition (I%) towards cell lines after 48 h treatment. Each dot represents mean ± SD (A549, MCF7, BJ-5ta, n = 3; HeLa, Raji, n = 4). (**B**) The changes in cell morphology of different cell lines induced by KTt-45 after 48 h treatment. The scale bars indicate 50 μm.

To determine anticancer activity of KTt-45, cytotoxicity assay was conducted on several cancer cell lines including A549 lung adenocarcinoma, HeLa cervical carcinoma, MCF-7 breast carcinoma, Raji Burkitt’s lymphoma. The results showed that at the concentration of 120 μM, the growth inhibition percentages were greater than 80% towards all cancer cell lines (Fig. 1A). To be more specific, these values reached 88.1%, 90.3%, 93.9%, and 100% for A549, HeLa, MCF-7, and Raji cells, respectively. Meanwhile, interestingly, BJ-5ta normal human fibroblasts seemed to be less sensitive to KTt-45 than cancer cells with 46.5% inhibition at 120 μM (Fig. 1A). The percentage inhibition remained high towards HeLa (72.8%) and Raji (60.6%) cells at 60 μM, but it considerably decreased to less than 30% for A549 and MCF-7 cells. At concentrations lower than 30 μM, the compound exhibited no toxicity to experimental cell lines. Regarding cell morphology induced by KTt-45, intriguingly and remarkably, the accumulation of intracellular vacuoles was observed inside HeLa cells at concentrations of 30 and 60 μM (Fig. 1B). The similar phenomenon also appeared on A549 cell line but it happened at higher concentrations (60 and 120 μM) (Fig. 1B). However, for Raji and MCF-7 cells, it seemed to be that these cells experienced a rupture of plasma membrane leading to cell lysis after KTt-45 treatment (Fig. 1B), which showed the diverse effects of the compound on different cancer cell lines.

As indicated in Table 1, IC_50_ values reached 73.8, 37.4, 73.2, and 56.0 μM towards A549, HeLa, MCF-7, and Raji cell lines, respectively. Notably, the compound displayed highly selective cytotoxicity towards HeLa cells when compared with normal fibroblasts, with selectivity index (SI) greater than 3.2 (Table 1). Therefore, due to the selectively cytotoxic property accompanied by the notable effect on cell morphology, the anticancer activity of KTt-45 compound against HeLa cell line was subjected to further investigation.

**Table 1:** The IC_50_ and SI values of KTt-45 on cell lines.

Cell line	IC_50_ (μM)		Selectivity index (SI) of KTt-45
	KTt-45	DOX	
A549	73.8 ± 10.4	5.9 ± 3.4	> 1.4
HeLa	37.4 ± 8.6	0.45 ± 0.05	> 3.2
MCF-7	73.2 ± 10.9	2.9 ± 2.2	> 1.5
Raji	56.0 ± 8.4	0.3 ± 0.07	> 2.6
BJ-5ta	> 120	3.0 ± 1.2	

The values display mean ± SD (A549, MCF7, BJ-5ta, n = 3; HeLa, Raji, n = 4). *DOX* doxorubicin.

### Effect of KTt-45 on HeLa cell growth

**Figure 2:**
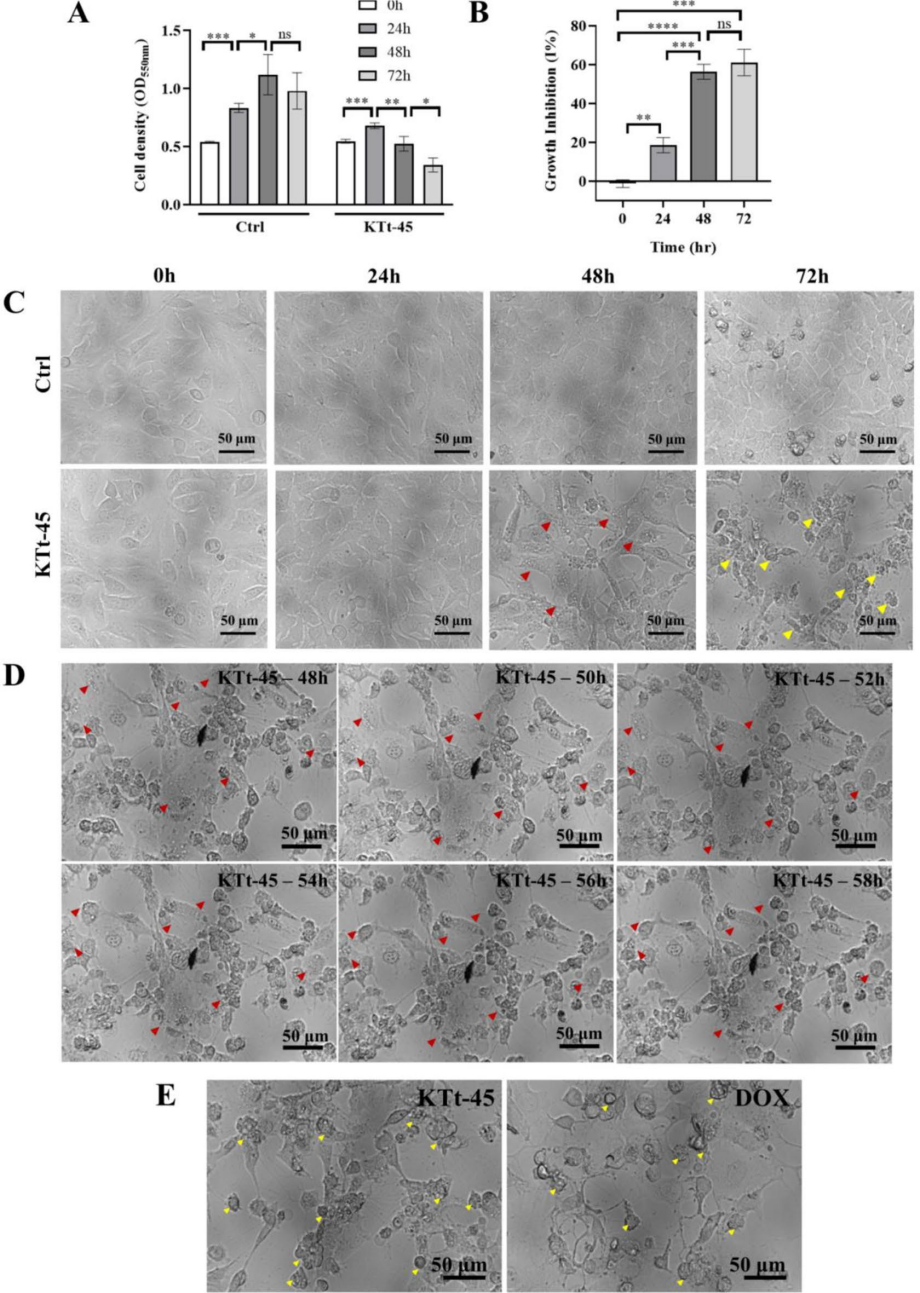
The effect of KTt-45 on HeLa cell growth and cell morphology during time. (**A**) The cell density (OD_550nm_) of samples treated with KTt-45 (37.4 μM) at different time points in comparison with control untreated samples (0.1% (v/v) DMSO). (**B**) The percentage growth inhibition of KTt-45 towards HeLa cell line during time. Each value represents mean ± SD (n = 3). The statistical differences were analyzed by two-tailed Student’s *t* tests (*p ≤ 0.05, **p ≤ 0.01, ***p ≤ 0.001, ****p ≤ 0.0001, ns, p > 0.05). (**C**) The cell morphology of HeLa cells incubated with KTt-45 (37.4 μM) at different time points. (**D**) The morphological changes of specific vacuole-containing HeLa cells induced by KTt-45 from 48 to 58 h. (**E**) The morphology of HeLa cells treated with KTt-45 (37.4 μM) in comparison with those treated with doxorubicin (3 μM) after 58 h incubation. The scale bars indicate 50 μm. The red arrows point cells containing several cytoplasmic vacuoles. The yellow arrows point shrunken cells with membrane blebbing. *DOX* doxorubicin.

To assess the effect of KTt-45 on cell growth, the cell density expressed by OD_550nm_ value was monitored every 24 h. As shown in Fig. 2A, for the group treated with KTt-45, cell density slightly increased at 24 h before significantly reducing by 2 times in the period from 24 to 72 h. However, the OD_550nm_ value of treated group at 24 h was lower than that of control untreated group, proving KTt-45 started to express growth inhibitory ability at this time point prior to a lethal effect on HeLa cells after 48 h. Meanwhile, for control group, the cell density continuously went up by 2.1 times from 0 to 48 h before it witnessed a 1.1-fold decline, which might be due to the high confluence. Besides, the percentage growth inhibition substantially rose during experimental time and reached the highest level of 61.1% at 72 h (Fig. 2B).

Regarding cell morphology, while untreated cells attached to culture surface and expressed epithelial appearance, KTt-45-treated ones showed abnormal morphological changes. Specifically, at 48 h, numerous cytoplasmic vacuoles appeared inside cells of treated group (Fig. 2C). After that, at 72 h, the majority of cell population began to round and shrink, accompanied by membrane blebbing phenomenon (Fig. 2C). To discover whether the accumulation of intracellular vacuoles at 48 h and the shrunken morphology at 72 h were two separate responses of different cell populations under KTt-45 treatment, we followed the cell appearance in the period from 48 to 72 h. As illustrated in Fig. 2D, the cells containing cytoplasmic vacuoles, which were observed at 48 h, gradually transformed into shrunken cells within 6 to 10 h. Therefore it might be that those morphological features belonged to consecutive stages of HeLa cells in response to KTt-45 treatment. Furthermore, since the shrinkage of cell and the cell membrane blebbing were similar to apoptotic characteristics previously described^28^, in this study, doxorubicin, which is a well-known apoptotic inducer^29^, was utilized to observe the changes of HeLa cells in apoptotic cell death. Interestingly, both doxorubicin-treated and KTt-45-treated groups shared identical morphological features (Fig. 2E). These results suggested that KTt-45 induced apoptosis in HeLa cervical carcinoma cells. Therefore, next, we evaluated different features of apoptosis including nuclear morphology and the activation of caspase enzymes.

### Assessment of cell nuclear morphology

**Figure 3:**
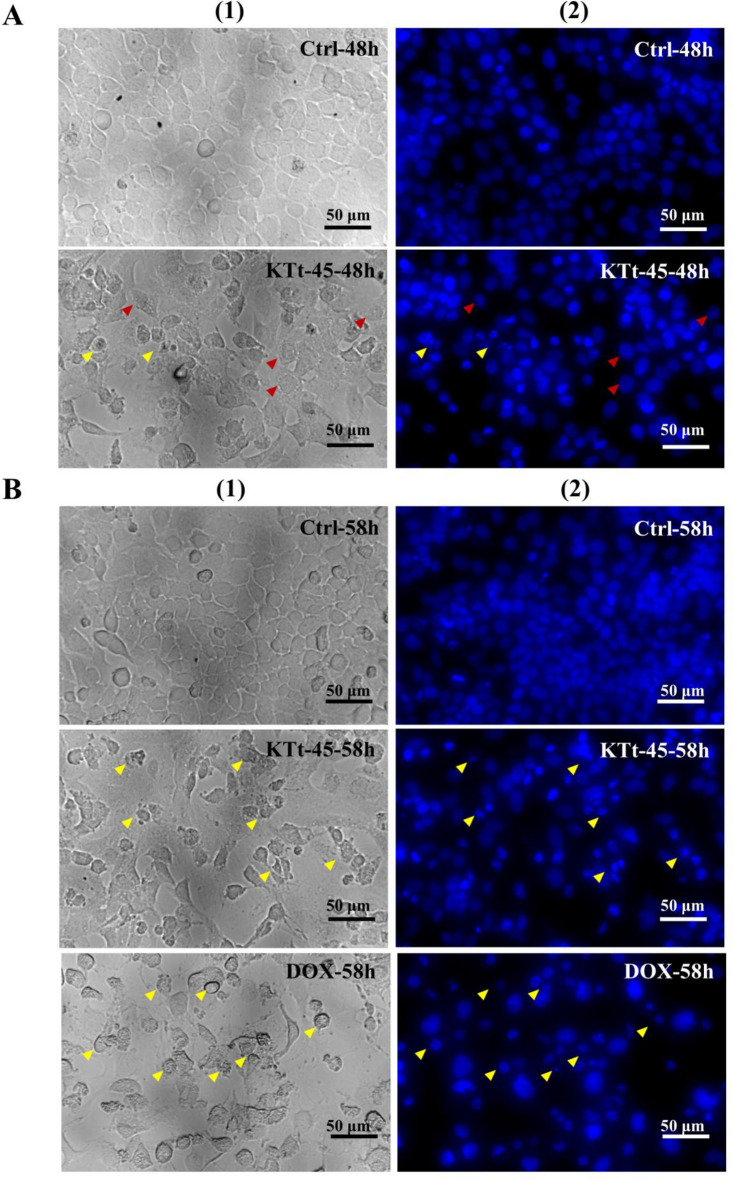
The changes in HeLa cell nuclei induced by KTt-45. (**A**) The nuclei of KTt-45-treated cells (37.4 μM) compared with control untreated cells (0.1% (v/v) DMSO) at 48 h. (**B**) The nuclei of KTt-45-treated cells (37.4 μM) compared with doxorubicin-treated (3 μM) and control untreated cells (0.1% (v/v) DMSO) at 58 h. Columns (1), (2) display images of cells (bright field) and cell nuclei (fluorescence field). The red arrows point vacuole-containing cells. The yellow arrows point shrunken cells. The scale bars indicate 50 μm.

DAPI, a DNA-binding dye, was utilized to visualize the nuclear morphology of KTt-45-treated group under a fluorescence microscope. As shown in Fig. 3A, at 48 h, the treated cells containing cytoplasmic vacuoles possessed normal nuclei similar to control untreated cells. Notably, nuclear disintegrity appeared in a scattered manner at this time point (Fig. 3A). Interestingly, after 58 h treatment, the number of fragmented and condensed nuclei significantly increased in the cell population induced by KTt-45 (Fig. 3B). Importantly, the nuclear morphology of group treated with KTt-45 was comparable to that of doxorubicin-treated one. It is documented that apoptotic cell death exhibit chromatin condensation and nuclear fragmentation into apoptotic bodies^30^; therefore, the DAPI-staining results supported the possibility that KTt-45 triggered apoptosis in HeLa cells.

### Investigation of caspase-3 activation

**Figure 4:**
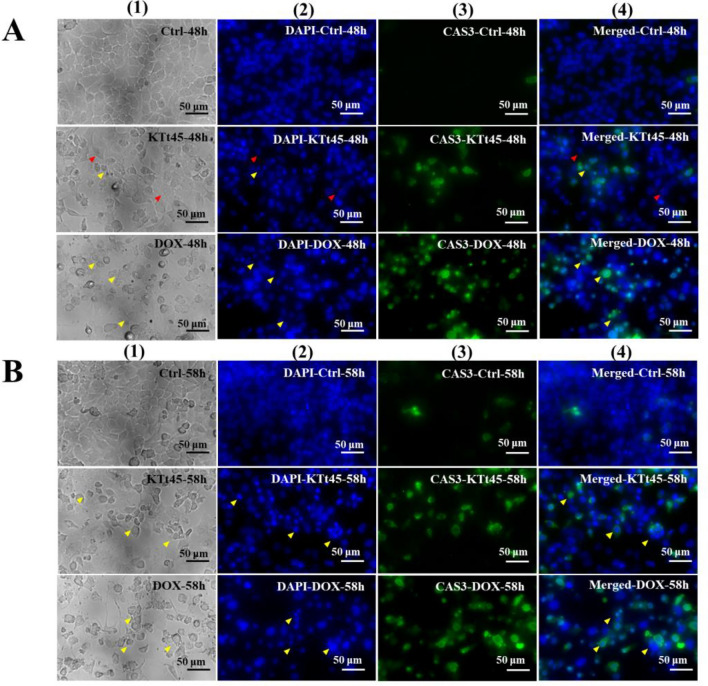
The caspase-3 activation in HeLa cells induced by KTt-45. (**A**) The cleaved-caspase-3 signals of KTt-45-treated cells (37.4 μM) compared with doxorubicin-treated (3 μM) and control untreated cells (0.1% (v/v) DMSO) at 48 h. (**B**) The cleaved-caspase-3 signals of KTt-45-treated cells (37.4 μM) compared with doxorubicin-treated (3 μM) and control untreated cells (0.1% (v/v) DMSO) at 58 h. Columns (1), (2), (3), and (4) display images of cells (bright field), cell nuclei (blue field), activated caspase-3 (green field), and merging (blue and green field), respectively. The red arrows point vacuole-containing cells. The yellow arrows point shrunken cells. The scale bars indicate 50 μm.

The activation of apoptotic cell death is considered as a target of anticancer agents due to not causing inflammation which could damage the organ and adjacent tissues. Caspase-3, synthesized as pro-enzyme, is cleaved, activated, and contributed as a main executor enzyme in apoptosis process^31,32^. Therefore, the activation of caspase-3 is considered as an important marker of apoptosis^33^. To examine whether apoptosis occurred in KTt-45-treated cells, immunofluorescence staining using anti cleaved-caspase-3 antibody was conducted. The captured images revealed that cleaved-caspase-3 signals significantly appeared in KTt-45-treated groups at both 48 h and 58 h (Fig. 4A,B). In addition, vacuole-containing cells expressed no signal (Fig. 4A). Interestingly, same as doxorubicin-treated population, the activation of caspase-3 was clearly present in KTt-45-treated cells at the location of shrunken cells owning condensed nuclei (Fig. 4B). So, taken together, KTt-45 could trigger apoptosis in HeLa cells through caspase-3 activation.

### Examination of caspase-9 activation

**Figure 5:**
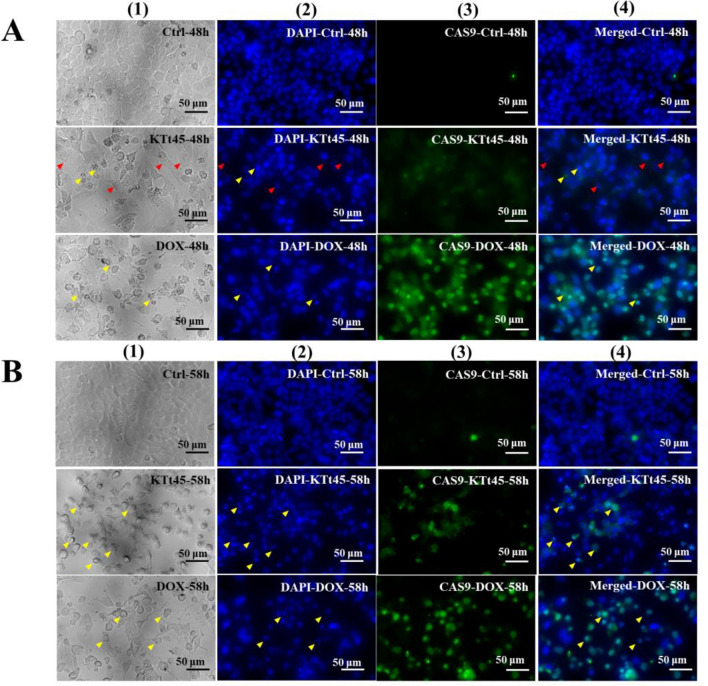
The caspase-9 activation in HeLa cells induced by KTt-45. (**A**) The cleaved-caspase-9 signals of KTt-45-treated cells (37.4 μM) compared with doxorubicin treated (3 μM) and control untreated cells (0.1% (v/v) DMSO) at 48 h. (**B**) The cleaved-caspase-9 signals of KTt-45-treated cells (37.4 μM) compared with doxorubicin treated (3 μM) and control untreated cells (0.1% (v/v) DMSO) at 58 h. Columns (1), (2), (3), and (4) display images of cells (bright field), cell nuclei (blue field), activated caspase-9 (green field), and merging (blue and green field), respectively. The red arrows point vacuole-containing cells. The yellow arrows point shrunken cells. The scale bars indicate 50 μm.

Apoptosis can be activated by extrinsic pathway through death receptors on cell membrane, and/or intrinsic pathway through mitochondria^34^. Regarding extrinsic pathway, when death ligands bind to Fas/TNF/TRAIL receptors, caspase 8/10 is processed and activate caspase 3 to initiate apoptosis. Meanwhile, in terms of intrinsic pathway, under DNA damage or cellular stress, cytochrome c from mitochondria would be released into cytoplasm to create apoptosome which cleaves caspase-9 prior to caspase-3 activation. To discover whether apoptosis is activated via mitochondrial pathway, the activation of caspase 9 was examined using anti cleaved-caspase-9 antibody. The results showed that caspase-9 activation occurred dispersedly at 48 h; in addition, at sites of the vacuole-containing cells, no signal was observed (Fig. 5A). Interestingly, caspase-9 activation appeared more remarkably at 58 h (Fig. 5B). Accordingly, apoptotic deaths in HeLa cells were triggered by KTt-45 through mitochondrial signaling pathway.

### Analysis of Annexin-V-FITC/PI

**Figure 6:**
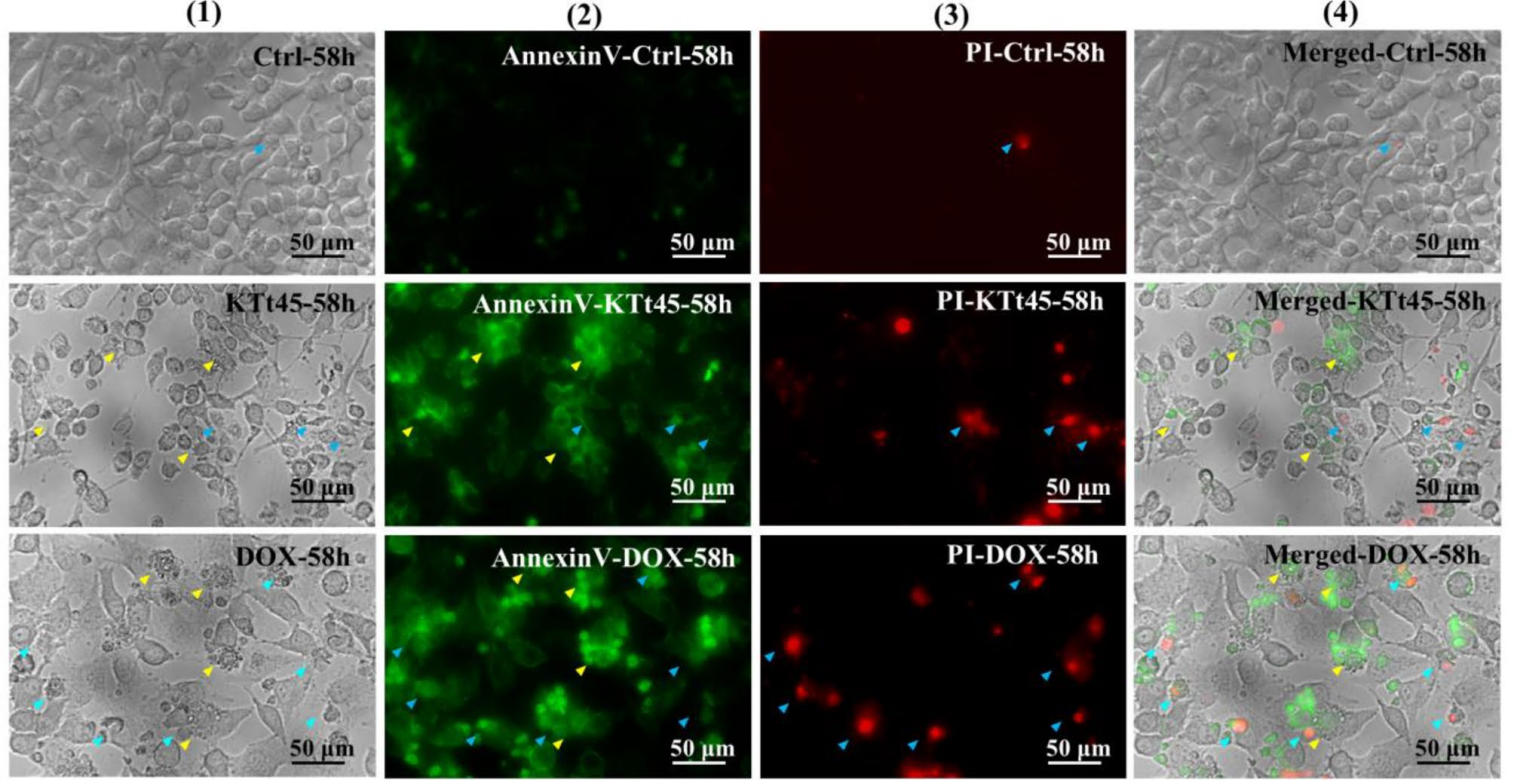
The analysis of annexin-V-FITC/PI in HeLa cells induced by KTt-45. The annexin-V and PI signals of KTt-45-treated cells (37.4 μM) compared with doxorubicin-treated (3 μM) and control untreated cells (0.1% (v/v) DMSO) at 58 h. Columns (1), (2), (3), and (4) display images of cells (bright field), annexin-V-FITC signals (green field), PI signals (red field), and merging (bright, green, and red field), respectively. The yellow arrows point early apoptotic cells. The blue arrows point late apoptotic/necrotic cells. The scale bars indicate 50 μm.

To confirm the apoptosis cell death, the analysis of annexin-V/PI is commonly utilized based on the fact that phosphatidylserine (PS) which is regularly located on inner cell membrane would be relocated to outer membrane in the early phase of apoptosis. Besides, propidium iodide (PI) which is a nucleic-binding dye could stain cell nuclear in case of dead cells with ruptured membranes implying late apoptotic/necrotic (annexin-V( +), PI( +)) stage, but expose no signal in case of the early apoptosis (annexin-V( +), PI(–)) or live cells (annexin-V(–), PI(–)). As shown in Fig. 6, annexin-V( +)/PI(–) signals appeared in shrunken, membrane-blebbing cells of both KTt-45- and DOX-treated samples, while annexin-V( +)/PI( +) signals were observed in extremely shrunken cells. Notably, the number of early-apoptotic cells occupied the major account in the cell population at 58 h after KTt-45 incubation. Besides that, there were no signals of annexin-V-FITC and PI in untreated samples. These results helped to re-confirm the occurrence of apoptotic cell death in HeLa cells under KTt-45 treatment.

### Investigation of autophagic vacuoles by MDC staining

**Figure 7:**
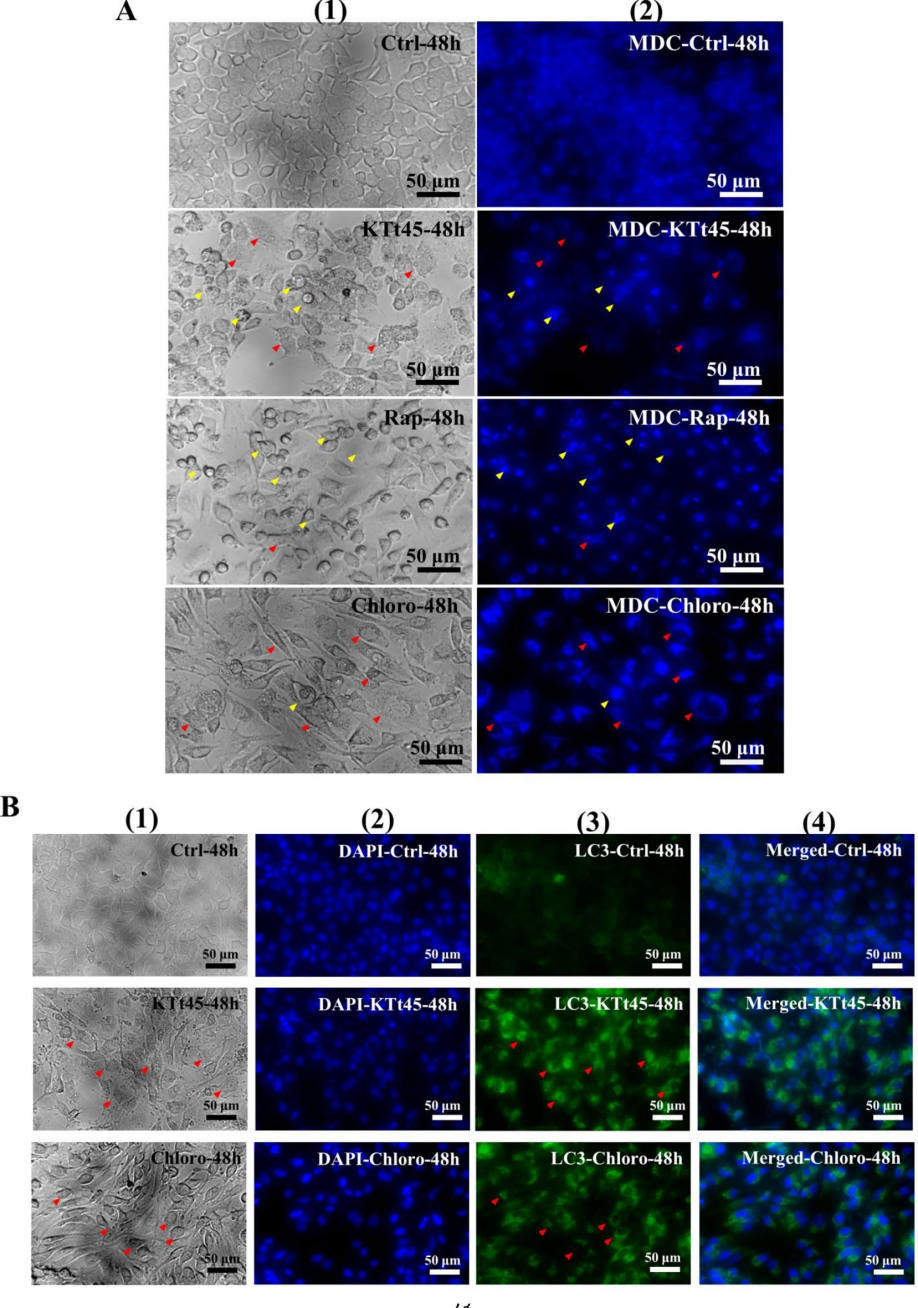
The autophagic markers’ staining of HeLa cells treated with KTt-45 at 48 h. (**A**) MDC staining results of control untreated (0.1% (v/v) DMSO), KTt-45-treated (37.4 μM), rapamycin-treated (40 μM), and chloroquine-treated (25 μM) cells; Columns (1) and (2) display images of cells (bright field) and MDC’s fluorescence (blue field). (**B**) LC3-staining results of control untreated (0.1% (v/v) DMSO), KTt-45-treated (37.4 μM), and chloroquine-treated (25 μM) cells; Columns (1), (2), (3), and (4) display images of cells (bright field), cell nuclei (blue field), accumulated LC3 signals (green field), and merging (blue amd green field) respectively. The red arrows point vacuole-containing cells. The yellow arrows point shrunken cells. The scale bars indicate 50 μm.

Regarding the accumulation of cytoplasmic vacuoles, the probability that autophagy was induced with KTt-45 treatment was examined by monodansylcadaverine (MDC) staining. Monodansylcadaverine (MDC) is an autofluorescence compound that specifically stains autophagosomes and autolysosome. Therefore, MDC staining is considered one of methods to recognize autophagic vacuoles^35^. In this assay, rapamycin, a well-known autophagy inducer^36^, was utilized for comparison. Besides, chloroquine, known as autophagy inhibitor due to inhibiting the fusion between autophagome and lysosome leading to the intense accumulation of autophagy vacuoles^37^, was also used. As shown in Fig. 7A, for KTt-45-treated cells, MDC-labeled vesicles were observed in vacuole-containing cells at 48 h, and MDC’s fluorescence increased immensely inside the shrunken cells, while the fluorescence signals also appeared in rapamycin-treated and chloroquine-treated samples (Fig. 7A). Accordingly, it is suggested that autophagic process was induced in HeLa cells after 48 h under KTt-45 treatment.

### Investigation of intracellular LC3 accumulation

LC3 is considered as one of markers of autophagy, in which following the autophagy induction, LC3 is cleaved by ATG4 family proteins to form LC3-I, then conjugated to phosphatidylethanolamine to create LC3-II localized on autophagosome’s membranes^38^. Due to the fact that LC3-II is degraded after the fusion between autophagosome and lysosome as normal protein turnover, chloroquine inhibiting this fusion could cause an accumulation of autophagosomes accompanied by the over-existence of LC3-II proteins in cytoplasm^37^. As shown in Fig. 7B, both KTt-45-treated and chloroquine-treated samples exhibited LC3 signals more intensely when compared with untreated control samples after 48-h incubation. Interestingly, the bright dots appeared in the same locations with vacuole-containing cells. Thus, these results supported the hypothesis that autophagy was triggered inside HeLa cells under KTt-45 treatment.

### Effect of KTt-45 on mRNA expression of HeLa cells

**Figure 8:**
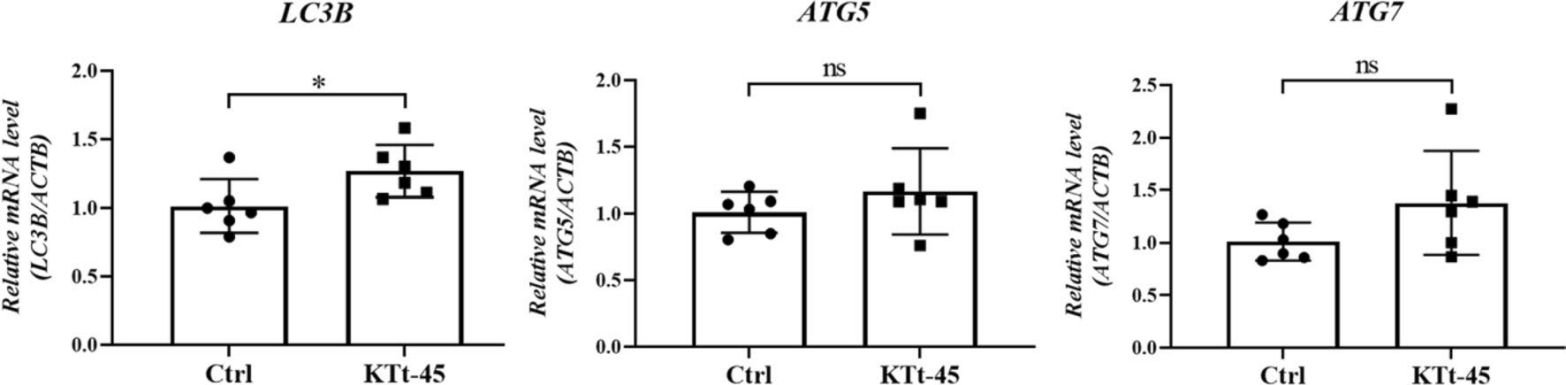
The relative mRNA expression level of HeLa cells treated with KTt-45 (37.4 μM). Each value represents mean ± SD of biological replicates (n = 6). The statistical differences were analyzed by two-tailed Student’s *t* tests (*p ≤ 0.05, ns, p > 0.05).

As described above, KTt-45 induced an accumulation of cytoplasmic vacuoles after 48 h incubation. Therefore, to investigate whether KTt-45 affected the expression of regulatory genes, relative mRNA levels of genes related to autophagic process (*ATG5, ATG7,* and *LC3B*) were investigated. The ATG5, ATG7 proteins contribute to membrane development of autophagic vacuoles in initial autophagic steps^39^. LC3B in the conjugation with phosphatidylethanolamine (PE) associating to autophagosome, is considered as a marker of autophagy^38^. The results showed that *LC3B* mRNA level increased by 1.25 times (Fig. 8). However, there were no changes in mRNA expression levels of *ATG5*, *ATG7* genes (Fig. 8). Thus, qRT-PCR results consolidated the hypothesis that autophagy was triggered inside HeLa cells under KTt-45 treatment.

## Discussion

In this study, the results showed that KTt-45 caused toxic effects on HeLa cervical, Raji lymphoma, MCF7 breast, and A549 lung cancer cell lines with IC_50_ values of 37.4, 56.0, 73.2, and 73.8 μM, respectively. For an in vitro bioassay, a pure compound with IC_50_ value less than 20 μM is considered as owning high toxicity, while IC_50_ ranging from 20 to 100 μM indicates a moderate activity; an inactive compounds display IC_50_ values greater than 200 μM^40^. Accordingly, KTt-45 compound showed anticancer ability towards all investigated cancer cell lines. In line with other studies, several compounds including 6-PNG, kushenol E, bonannione A, and nymphaeol A which share similar structural characteristics have been reported for IC_50_ values from 1 to 100 μM on varied types of cancer including melanoma, prostate, colorectal, breast, cervical, ovary, and lung cancer^3,4,6,7,41–43^.

KTt-45 showed the lowest IC_50_ value of 37.4 μM towards HeLa cells, and more importantly, the compound caused less toxicity to normal fibroblast BJ-5ta cells (IC_50_ > 120 μM) when compared with doxorubicin (IC_50_ = 3 μM on BJ-5ta cells). In previous documents, selectivity index (SI) was also utilized to monitor selective effect of a compound on cancer cells compared with normal cells, in which SI ≥ 3 points out a prospective sample^44^. Thus, KTt-45 compound exhibiting a highly selective cytotoxicity against HeLa cervical adenocarcinoma cell line (IC_50_ = 37.4 μM, SI > 3.2) becomes a potential agent for further investigation.

Regarding the anticancer mechanism of KTt-45, the specific effects on HeLa cell morphology induced by the compound were also observed. Particularly, numerous intracellular vacuoles appeared in KTt-45-treated cells after 48 h incubation, then the cells became round, shrunken with membrane blebbing leading to cell deaths as evidenced by a significant decrease in OD_550nm_ value at 72 h. The analytical results showed that round shrunken cells experienced chromatin condensation, nuclear fragmentation, the cleavage of caspase-3 as well as caspase-9, and the positive staining of annexin-V-FITC which proved the activation of mitochondrial-dependent apoptosis in HeLa cells. In relation to other works, surprisingly, 6-PNG expressed non-apoptotic activity on SK-MEL-28 melanoma^7^, PC3 and DU145 prostate cancer cell lines^6^. Interestingly, the accumulation of cytoplasmic vacuoles also appeared in PC3 and DU145 cells after 12 h incubation with 6-PNG^6^, which is similar to the phenomenon in our study except for non-apoptotic deaths. Thus, compared with 6-PNG, the apoptotic activation activity of KTt-45 which was newly observed in the current study could be explained by the differences in investigated cancer cell lines or related to the modification in the KTt-45’s skeleton including the transfer of hydroxyl group from 4’ to 2’ site (Supplementary Fig. S2). In a previous document, the 2'-OH group performed the most potent cytotoxic effect among 2’-OH, 4’-OH, 6-OH, and 7-OH groups on colorectal cancer cells with the mechanism causing an elevation of reactive oxygen species (ROS) and apoptosis activation^45^. In addition, kushenol E, which possesses similar chemical skeleton to KTt-45 with 2’-OH group, could trigger apoptosis and inhibit autophagosome-lysosome fusion in HeLa cells^42^. Besides, as shown in Supplementary Fig. S2, the replacement of prenyl (3″, 3″-dimethylallyl) group of 6-PNG by 3″, 3″-diethylallyl group of KTt-45 is a notable property although its effect on anticancer activity has not been reported. It is also documented that several T-type calcium channel blockers could trigger mitochondrial apoptosis by caspase-9 activation in different cancer cell lines^19,21^. However, despite being a T-type calcium channel blocker^8^, KTt-45’s effect on intracellular calcium level of HeLa cells in relationship with anticancer ability still need to be elucidated in further studies.

In terms of the appearance of intracellular vacuoles, the configuration is the same as autophagic vacuoles illustrated in former studies^46,47^. Our MDC-, LC3-staining, and qRT-PCR results supported the possibility that autophagy process occurred in HeLa cells under KTt-45 treatment. Autophagy is characterized by the accumulation of intracellular vacuoles which are formed by the sequestration of cytoplasmic portion into a double membrane organelle called autophagosome; then the autophagosome fuses with lysosome to become autolysosome which degrades and recycles cellular contents^48^. In normal condition, autophagy plays pro-survival role, in which this process helps to recycle intracellular constituents such as long-lived proteins, aged or damaged organelles to provide energy and maintain cell homeostasis in response to starvation, hypoxia, metabolic stress, pathogen infection, and chemotherapeutic intervention^48^. It is also reported that autophagy contributes to tumor progression as well as therapeutic resistance due to autophagy’s cytoprotective role against different types of stress^49,50^. Thus, autophagy inhibitors which could enhance the effectiveness of chemotherapeutic agents and sensitize chemotherapy-resistant cells become potent candidate for cancer treatment research^49,51^. In our study, under KTt-45 treatment, the accumulation of vacuoles happened prior to apoptosis, which is predicted that autophagy preceded apoptosis. This phenomenon was also observed in a previous work in which the appearance of autophagic vacuoles were induced before apoptotic cell death^52^. In addition, mibefradil, a T-type calcium channel blocker, could also induce apoptotic cell death preceded by ER stress and autophagy inhibition^19^. Since the accumulation of intracellular autophagic vacuoles could be related to the increase in autophagy induction or the inhibition of autophagosome-lysosome fusion, in the current study, whether the appearance of cytoplasmic vacuoles suggested as autophagic vacuoles was a cell response against stresses exerted by KTt-45 or autophagy-inhibitory effect of KTt-45 remains undetermined. Therefore, to elucidate more in-depth mechanism, the relationship between autophagy process and apoptotic activation, as well as the effect on Ca^2+^ intracellular concentration in connection with cell death under KTt-45 treatment still has to be elucidated in next studies.

Importantly, KTt-45 pushed cancer cells towards apoptotic deaths at last, proving the potential of KTt-45 in cancer treatment research. For drug development in the future, KTt-45 could be used as a single agent or in combination with other chemotherapeutic drugs in order to enhance treatment efficacy, cope with the current chemotherapeutic resistance, as well as relieve neuropathic pain for cancer patients.

## Conclusions

In a previous study, the novel synthetic compound KTt-45 (6-(3-ethylpent-2-enyl)-5,7-dihydroxy-2-(2-hydroxyphenyl)chroman-4-one) proved the ability in restoring neuropathic pain induced by partial sciatic nerve ligation and reversing bortezomib-induced peripheral neuropathy in multiple myeloma thank to the inhibitory ability of T-type calcium channels. Due to the fact that calcium signaling also contributes to tumor growth, the current study conducted an investigation of anticancer activity of KTt-45 on various human cancer cell lines. The results revealed that KTt-45 caused cytotoxic effects towards HeLa cervical cancer, Raji lymphoma, MCF-7 breast cancer, and A549 lung cancer cell lines with IC_50_ values less than 100 μM. Especially, for HeLa cells, KTt-45 exhibited more selective toxicity (IC_50_ = 37.4 μM, SI > 3.2) when compared with BJ-5ta normal human fibroblasts. In respect of mechanism, KTt-45 inhibited HeLa cell’s growth, induced an accumulation of cytoplasmic vacuoles after 48 h, and activated mitochondrial-dependent apoptosis later. Thus, KTt-45 is a potential novel agent in cancer treatment research.

### Supplementary Information

Supplementary Figures.

## Data Availability

All data generated or analyzed during this study are included in this published article and its supplementary information files. The raw data is available on request from the corresponding author.

## Abbreviations

6-PNG: 6-Prenylnaringenin
Ctrl: Control
DOX: Doxorubicin
IC_50_: 50% Growth inhibitory concentration
KTt-45: 6-(3-Ethylpent-2-enyl)-5,7-dihydroxy-2-(2-hydroxyphenyl)chroman-4-one
SI: Selectivity index
OD_550nm_: Optical density at wavelength 550 nm
