# Supplementary material for: KTt-45, a T-type calcium channel blocker, acts as an anticancer agent by inducing apoptosis on HeLa cervical cancer cell line
Source: Sci Rep. 2023 Dec 13;13:22092. doi: 10.1038/s41598-023-47199-1 (PMC10716508; doi:10.1038/s41598-023-47199-1)
Supplement: Supplementary file 1 — Supplementary Figures. [file 41598_2023_47199_MOESM1_ESM.pdf]

Investigating the Anticancer Potential of KTt-45: A T-Type Calcium Channel Blocker in Human Cancer Cell Lines

Nguyen Huy Du, Truong Thi Bich Ngoc, Huynh Qui Cang, Nguyen Thi Thuy Luyen, Tran Linh Thuoc, Tran Le Quan, Dang Thi Phuong Thao

Figure S1

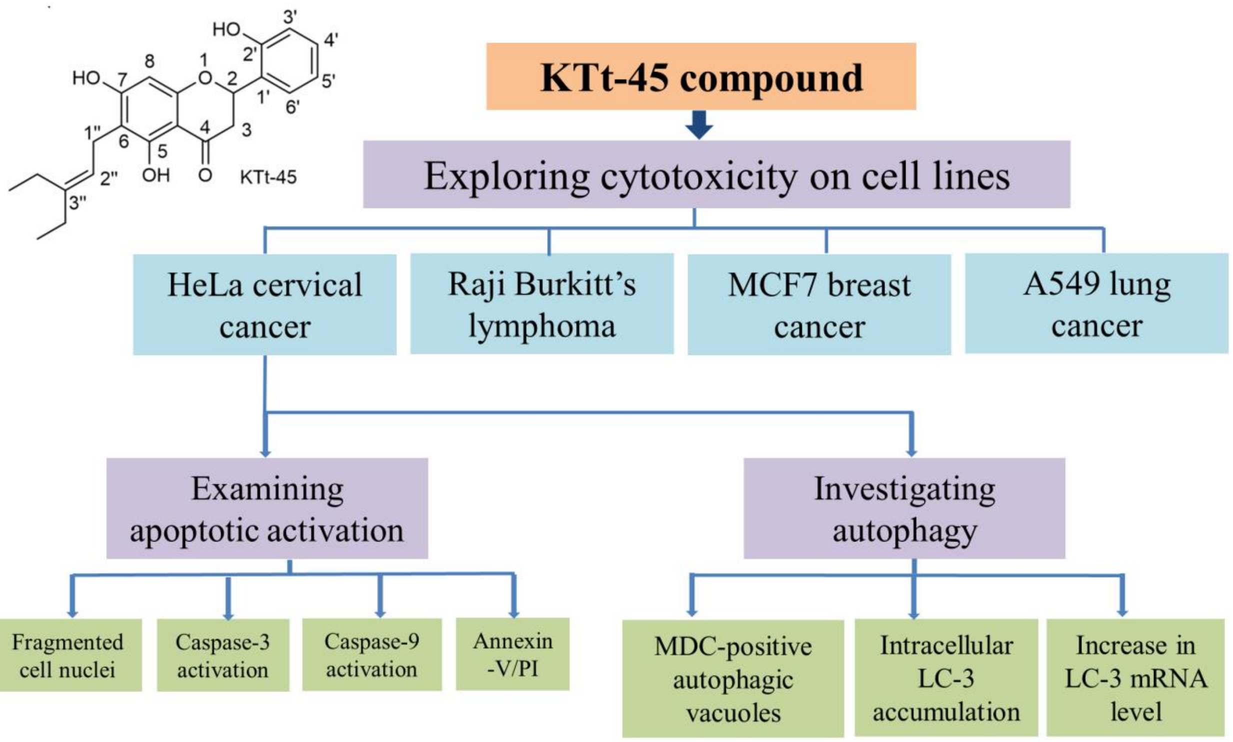

Figure S1. Schematic diagram of the experimental design.

Figure S2

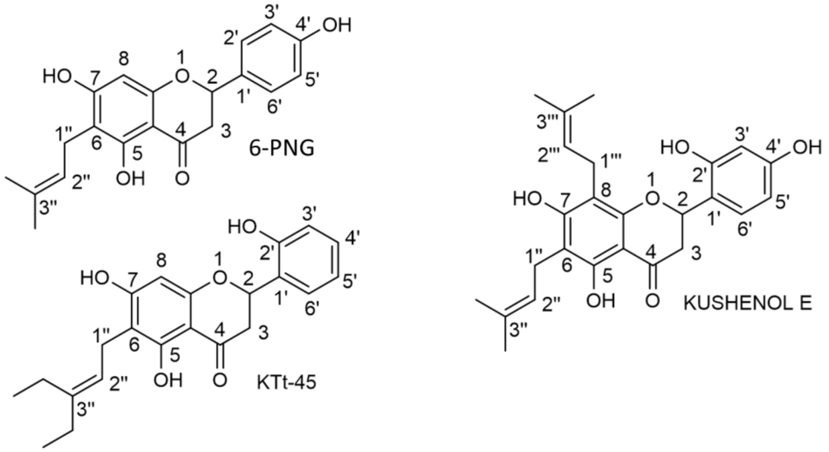

Figure S2. The chemical structure of KTt-45, 6-prenylnaringenin (6-PNG), and kushenol E.
